# Leadership Styles and Delegation Practices of Head Nurses as Perceived by Senior Staff Nurses at a Hospital in Bangladesh

**DOI:** 10.1155/jonm/5184762

**Published:** 2025-09-18

**Authors:** Md. Golam Kibria

**Affiliations:** Department of Medical and Surgical Nursing, Grameen Caledonian College of Nursing, Dhaka, Bangladesh

**Keywords:** delegation, leadership style, nursing management

## Abstract

**Background:** Effective leadership and delegation are essential in nursing management to improve patient care outcomes. However, there is limited research on the relationship between leadership styles and delegation practices in the South Asian context.

**Objective:** To examine the relationship between leadership styles and delegation practices of head nurses as perceived by senior staff nurses at a hospital in Bangladesh.

**Methods:** A descriptive correlational design was used with 112 senior staff nurses at Shaheed Suhrawardy Medical College Hospital, Dhaka, Bangladesh. Data were collected using the Delegation Questionnaire and the MLQ 5X-short (Cronbach's α > 0.78). Pearson's correlation assessed relationships between leadership styles and delegation.

**Results:** Most head nurses were perceived as practicing transformational leadership (mean = 25.17), followed by transactional (12.97) and laissez-faire (5.66). Delegation was rated as “sometimes” by 59.8% of the respondents, with significant positive correlations between delegation and transactional leadership (*r* = 0.321, *p* < 0.01) and weaker positive correlations with transformational leadership (*r* = 0.206, *p* < 0.05).

**Conclusion:** Transformational leadership was most common, but correlations with delegation were weak to moderate, indicating statistical but limited practical significance. Findings provide baseline evidence for leadership training and policy development in nursing leadership in Bangladesh.

## 1. Introduction

Leadership and delegation are crucial elements of nursing management, directly impacting team performance, staff satisfaction, and patient care outcomes. Effective delegation allows nursing staff to engage in meaningful tasks, enhance their clinical skills, and ensure optimal care delivery. However, the degree to which delegation occurs depends largely on the leadership styles practiced by nurse managers, particularly head nurses.

In the context of nursing, delegation is the process of transferring responsibility for specific tasks to another while retaining accountability for outcomes [1]. It is essential for the efficient functioning of healthcare teams, especially in resource-constrained environments like those in Bangladesh, where nurse-to-patient ratios are high and the demands on nursing staff are significant.

Healthcare organizations consider delegation for the achievement of job empowerment and loyalty. Nurses were highly perceived in all the dimensions of delegation skills (mean = 85.44). There was a positive, statistically significant correlation between nurses' perception of nurse manager delegation skills and their empowerment and loyalty (*p*=0.00). Hospital administrators should generate a strategic plan to improve nurses' empowerment and loyalty level, develop essential guidelines for delegation, and design educational programs for nurses about duties and instructions of delegated tasks [2].

A large percentage (66.7%) of the participants were not in agreement with a statement that nurse managers do not feel they have enough time to delegate properly. About 37.8% disagreed that they would delegate more if they had more encouragement and appreciation. About 64.4% of the study participants gave an agreement with the statement that they get upset when the delegated task is not done and is incomplete after a thorough delegation of instructions [3].

Leadership is the ability to mobilize, influence, and communicate the organizational mission and vision to motivate, empower, and inspire others to act willingly toward achieving organizational goals [4]. Effective leadership helps nurses understand the expected qualities for building relationships among nurses, interdisciplinary teams in the nursing profession, and patients. Some leadership qualities include appearance, self-confidence, self-awareness, motivation, empathy, honesty, vision and purpose, integrity, emotional intelligence, social skills and abilities, commitment, and passion. Others are humility, creativity and innovation, accountability, delegation, resilience, empowerment, and teamwork [5].

Leadership styles are generally divided into three main types: transformational, transactional, and laissez-faire. Transformational leadership, which involves motivation, inspiration, and intellectual stimulation, is often associated with higher job satisfaction and improved team performance [6]. Transactional leadership, by contrast, emphasizes structured tasks, clear expectations, and rewards, making it effective in high-pressure situations [7].

The laissez-faire style, which entails minimal intervention, has been criticized for its negative effects on team morale and job satisfaction [8]. Leadership styles that promote a positive work environment, such as transformational and democratic leadership, are linked to greater job satisfaction and higher retention rates among nurses [9].

Leadership is an inherent part of nursing practice because most nurses work in groups or units. Holding a registered nurse (RN) license indicates certain leadership abilities and involves the capacity to delegate and supervise others. Leadership can be seen as the ability to build confidence and support among followers, especially in organizations where competence and commitment lead to performance [10].

Transactional leadership styles were more commonly practiced by nurse managers, which indicated that nurse managers focus on maintaining the current situation, enforcing rules and procedures, and ensuring compliance. Nursing leaders must concentrate on developing transactional leadership skills to ensure patient safety and quality of care [11].

The nurses (56%) had a high laissez-faire style, 67.9% had moderate delegation, and 80.4% had moderate self-confidence. A significant positive correlation exists between delegation, authoritative, and democratic leadership styles. However, there was no correlation between leadership styles and self-confidence. Regular training courses for head nurses on effective leadership abilities and different leadership styles, as well as seminars on delegation and problem-solving. It also emphasizes the importance of teamwork and building trust in staff abilities to boost [12].

Head nurses (65.3%) sometimes delegated tasks, 21.2% always delegated, and transformational leadership was the most common style, followed by transactional and servant leadership, while authentic leadership was least used; it also revealed that autocratic head nurses often avoided delegation, whereas laissez-faire head nurses frequently delegated, leading to the recommendation for training on effective delegation and promoting respectful leadership styles like authentic, democratic, and servant leadership [13].

The majority of the respondents were aged less than 32 years (69.3%) with a mean of 1.31 ± 0.463 and females (80.4%) with a mean age of 32.56 ± 7.7 years. The average monthly income of the participant was 32,944.46 (7380.95) Taka. A greater proportion was educated with a Diploma in nursing (50.0%) and had taken in-service training, yes (29.5%), with several training services 0.63 (1.12). The mean leadership skill of clinical nurses was 3.51 (SD = 0.63). Their leadership skills were at a moderate level. The leadership skills were poor among the nurses. To improve the nurses' leadership skills, it is necessary to develop continuing in-service education and training regarding leadership skills for ensuring patient safety [14].

In Bangladesh, the nursing leadership landscape is underexplored, and the relationship between leadership styles and delegation practices is a limited study. This study seeks to fill this gap by exploring the perceptions of senior staff nurses regarding the leadership styles of head nurses and their impact on delegation practices.

This study aims to examine the relationship between leadership styles and delegation practices of head nurses as perceived by senior staff nurses at a hospital in Bangladesh. The specific aims were to (1) assess the degree of delegation practices of head nurses as perceived by senior staff nurses; (2) identify the leadership styles of head nurses as perceived by senior staff nurses; (3) examine the relationship between leadership styles and delegation practices of head nurses as perceived by senior staff nurses; and (4) find out the sociodemographic characteristics of senior staff nurses.

## 2. Methods

### 2.1. Study Design and Setting

A descriptive correlational design was adopted to examine the relationship between leadership styles and delegation practices of head nurses as perceived by senior staff nurses. The study was conducted at Shaheed Suhrawardy Medical College and Hospital (ShSMCH), a 1350-bed tertiary-level hospital in Dhaka, Bangladesh, employing approximately 691 nurses.

### 2.2. Study Subjects and Sampling

The study population comprised senior staff nurses with at least 1 year of clinical experience at ShSMCH. Using G∗Power 3.1.9.2, a minimum sample size of 84 was calculated (α = 0.05, power = 0.80, and effect size = 0.30). To account for potential attrition, 20% was added, resulting in a target sample of 112. A nonprobability convenience sampling technique was used. Exclusion criteria included nursing administrators (e.g., Nursing Superintendent, Deputy Nursing Superintendent, Nursing Supervisor, and Ward In-charge).

### 2.3. Data Collection Instruments

Data were collected using a structured questionnaire comprising the following three sections:

• Section 1: Sociodemographic Questionnaire: this section included questions about participants' age, gender, education, years of experience, unit, and leadership training.
• Section 2: Delegation Practices Questionnaire: this section featured a 30-item Likert scale (1 = *strongly disagree* to 5 = *strongly agree*), measuring perceived delegation by head nurses. Scores < 50% indicated “never delegate,” 50%–75% “sometimes delegate,” and > 75% “always delegate.”
• Section 3: Leadership Style Questionnaire: this section included the Modified Multiple Leadership Questionnaire (MLQ-5X short) [18], containing 21 items: transformational (12 items), transactional (6 items), and laissez-faire (3 items). Items were rated from 0 (“*not at all*”) to 4 (“*frequently, if not always*”).

Content validity was confirmed by three experts (CVI: 0.93 for delegation, 0.94 for leadership styles). Reliability testing yielded Cronbach's α = 0.83 for delegation and 0.80 for leadership styles. Pretesting with 11 nurses ensured cultural and contextual appropriateness.

### 2.4. Data Collection

Following institutional permission and informed consent, questionnaires were distributed in sealed envelopes to eligible nurses and collected within 3 days. Completion time was approximately 20–30 min. Anonymity was maintained through numerical coding.

### 2.5. Data Analysis

Data were analyzed using Statistical Package for the Social Sciences (SPSS) (Version 26). Descriptive statistics (frequency, percentage, mean, and SD) summarized demographic and study variables. Pearson's correlation coefficient assessed relationships between leadership styles and delegation practices. The normality of variables was checked using Shapiro–Wilk tests and histograms. Assumptions for Pearson correlation were met; therefore, Pearson correlation was used.

### 2.6. Ethical Considerations

Ethical approval was obtained from the Institutional Ethical Review Board of Enam Medical College (Ref. no. EMC/IERB/2024/04-3). ShSMCH did not have its own IRB; therefore, approval was sought from the principal investigator's affiliated institution. Written informed consent was obtained from all participants. Data confidentiality, anonymity, and the right to withdraw at any time were assured.

## 3. Results

Table 1 shows that the mean age of the participants was mean = 34.83 (SD ± 7.46). More than half (53.6%) of the participants belong to the age group of 30–40 years. Most of the (88.4%) participants were female. In terms of professional nursing education, 33% of the participants had a diploma in nursing. The majority (67%) of the participants had less than 10 years of working experience as a professional nurse. Regarding the current department, 31.3% of the participants had worked at the medicine department, followed by ICU (21.4%) and surgery (22.3%). Only a few (12.5%) of the participants had training in leadership and management.

Table 2 presents the distribution of head nurses according to their agreement on delegation as perceived by senior staff nurses. Their highest-rated behavior was “watching the time to end tasks” (mean = 4.10, SD ± 0.97). This was followed by “gives remarks upon task completion” (mean = 4.05, SD ± 0.73). In contrast, the lowest-rated item was “cannot take a vacation as work would collapse” (mean = 3.08, SD ± 1.18). The mean total delegation score for the scale was (mean = 107.77, SD ± 13.27).

Table 3 displays the distribution of head nurses based on their level of delegation as seen by senior staff nurses. Most head nurses “sometimes delegate” tasks, with 59.8%. A significant portion, 37.5%, was perceived to “always delegate.” However, a small group (2.7%) was identified as “never delegating.”

Table 4 shows the perceived leadership styles of head nurses by senior staff nurses. The results indicated that transformational leadership was the most common style, with a mean score of (mean = 25.17, SD = 7.31), followed by transactional (mean = 12.97, SD = 3.29) and laissez-faire leadership (mean = 5.67, SD = 2.12). The mean total leadership score for the scale was (mean = 43.81, SD ± 10.90).

Table 5 presents the Pearson's correlations for the associations between leadership styles and delegation practices of head nurses as perceived by senior staff nurses. The findings revealed that delegation was positively and significantly correlated with overall leadership style (*r* = 0.290, *p* < 0.01). The strongest specific correlation was found between delegation and transactional leadership (*r* = 0.321, *p* < 0.01). A moderate, positive correlation was also observed between delegation and laissez-faire leadership (*r* = 0.279, *p* < 0.01). A weaker but statistically significant correlation was observed between delegation and transformational leadership (*r* = 0.206, *p* < 0.05).

Table 6 presents the multiple linear regression examining how head nurses' transformational, transactional, and laissez-faire leadership styles predict delegation practices as perceived by senior staff nurses. The overall model was statistically significant, *F* (3, 108) = 4.75 and *p*=0.004, explaining 11.7% of the variance in delegation practices (*R*^2^ = 0.117). Among the predictors, transactional leadership style emerged as a significant positive predictor of delegation practices (β = 0.238, *p*=0.037, and 95% CI [0.009, 0.29]), indicating that higher use of transactional behaviors is associated with more frequent or effective delegation. Neither transformational leadership style (β = 0.008 and *p*=0.942) nor laissez-faire leadership style (β = 0.138 and *p*=0.269) significantly predicted delegation.

## 4. Discussion

This study aimed to explore the relationship between leadership styles and delegation practices as perceived by senior staff nurses in a tertiary hospital in Bangladesh. The findings indicate that transformational leadership was the most prevalent style among head nurses, followed by transactional leadership, with laissez-faire leadership being the least practiced. These results align with global trends, where transformational leadership is often seen as more effective in promoting staff engagement and fostering a positive work environment [15].

A significant positive correlation was observed between transactional leadership and delegation practices (*r* = 0.321, *p* < 0.01), suggesting that transactional leaders provide clear guidelines and expectations that facilitate task delegation. However, the weak-to-moderate correlation between transformational leadership and delegation (*r* = 0.206, *p* < 0.05) indicates that while transformational leaders are inspirational, their impact on delegation may be limited in contexts requiring strict task supervision and control.

The finding that laissez-faire leadership showed only a weak positive correlation with delegation (*r* = 0.279 and *p*=0.003) supports the view that minimal intervention and lack of direction may hinder effective delegation [7]. In the Bangladesh context, where resource constraints and high patient loads exist, this style might not provide the necessary structure for effective delegation and task management.

Interestingly, although transformational leadership was the most frequently practiced style, delegation was perceived as a moderate practice among senior staff nurses, with 59.8% of the respondents indicating that head nurses “sometimes” delegated tasks. This suggests that while transformational leadership is associated with motivation and team development, other factors, such as organizational culture, staffing levels, and workload, also play a role in the delegation process.

In this study, multiple regression analysis revealed that transactional leadership style emerged as a significant positive predictor of delegation practices (β = 0.238, *p*=0.037, and 95% CI [0.009, 0.29]) among the three leadership styles. Specifically, higher use of transactional behaviors, such as clarifying performance expectations and associating rewards with task completion, was associated with more frequent or effective delegation. This finding aligns with earlier research, indicating that transactional leadership can enhance task allocation and accountability by establishing clear guidelines and contingent reinforcement [16, 17].

Interestingly, transformational leadership, despite being positively correlated with delegation in bivariate analysis, did not remain a significant predictor in the multivariate model. This suggests that while inspirational and visionary qualities may foster motivation, they may not directly influence the structured distribution of tasks in clinical settings where operational clarity is paramount. Similarly, laissez-faire leadership was not a significant predictor, reflecting its generally passive approach and limited engagement in decision-making. Overall, the results highlight the importance of task-oriented, reward-linked leadership behaviors in optimizing delegation practices within hospital nursing management in the Bangladeshi context.

## 5. Implications for Nursing Management

The study's findings suggest that nursing management should prioritize leadership development programs that enhance both transformational and transactional skills, particularly those that support effective delegation. Standardized delegation guidelines, mentorship, and supportive staffing models are essential to reduce delegation barriers such as fear of losing control or overdependence on head nurses. Integrating delegation and leadership performance into appraisals and training can strengthen accountability and improve team efficiency, ultimately enhancing patient care and staff satisfaction.

## 6. Limitations and Future Research Directions

This study was conducted at a single institution in Dhaka, limiting its generalizability to other regions in Bangladesh or South Asia. Additionally, the study's reliance on self-reported perceptions introduces the possibility of response bias, as nurses may have been influenced by their personal views on leadership. Future studies should explore the impact of leadership training on delegation practices and job satisfaction in diverse healthcare settings. Hospital administrators should consider implementing leadership development programs focused on transformational and transactional leadership styles to foster a culture of effective delegation and improve overall team performance. Moreover, policymakers should develop guidelines to support head nurses in their delegation roles, ensuring that these practices are aligned with organizational objectives and patient care standards.

## 7. Conclusion

This study provides baseline evidence on the relationship between leadership styles and delegation practices of head nurses as perceived by senior staff nurses at a hospital in Bangladesh. The findings revealed that while transformational leadership was most commonly practiced, transactional leadership had a stronger association with delegation effectiveness. These results underscore the importance of strengthening leadership competencies through targeted training programs and establishing clear delegation protocols in clinical settings. By informing future leadership development initiatives and policy formulation, this study contributes valuable insight toward improving nursing management and organizational efficiency in the Bangladeshi healthcare context.

## Figures and Tables

**Table 1:** Distribution of sociodemographic characteristics of the participants (*N* = 112).

Variables	Frequency (*n*)	(%)	Mean ± SD
Age (years) (minimum: 25; maximum: 53)			34.90 ± 6.06
< 30	30	26.8	
30–40	60	53.6	
> 40	22	19.6	
Gender			9.79 ± 4.59
Male	13	11.6	
Female	99	88.4	
Working experience			
< 10	75	67.0	
10–20	32	28.6	
> 20	5	4.5	
Highest professional education			
Diploma in nursing	37	33.0	
BSc in nursing	14	12.5	
BSc in nursing (post basic)	24	21.4	
Master of science in nursing	13	11.6	
Master of public health	24	21.4	
Current department/unit			
Dialysis	9	8.0	
Gyne	9	8.0	
ICU	24	21.4	
Medicine	35	31.3	
Neonatology	5	4.5	
OT	5	4.5	
Surgery	25	22.3	
Training in leadership and management			
Yes	14	12.5	
No	98	88.5	

**Table 2:** Distribution of head nurses according to their agreement of delegation as perceived by senior staff nurses (*N* = 112).

Variables	Mean ± SD
Delegate tasks to ease the workload	3.35 ± 1.11
Show confidence in completing tasks	3.54 ± 0.94
Allow staff to undertake delegated work	3.60 ± 0.83
Know everyone's strengths and weaknesses	3.53 ± 1.01
More precise than others	3.54 ± 1.18
No one can work as a head nurse	3.41 ± 1.11
No one cares about work as the head nurse	3.47 ± 1.03
Do not trust anyone with work secrets	3.41 ± 1,14
Afraid that if the head nurse delegates someone, he/she will hate the head nurse	3.38 ± 1.23
Delegation of opportunity to develop individuals	3.79 ± 0.96
Provide training to inexperienced staff	3.63 ± 1.18
Supervise every detail of staff work	3.79 ± 0.96
Tell the staff their authority when delegating the task	3.60 ± 1.14
Cannot take a vacation, work will collapse	3.08 ± 1.18
Tolerate mistakes	3.32 ± 1.08
Balance the workload of the staff	3.55 ± 1.00
Watch the work of the staff and correct mistakes	3.66 ± 1.10
Find myself compelled to rework	3.41 ± 1.09
Delegate all jobs, review the results	3.81 ± 0.94
Staff lack commitment, if delegated, work will not be implemented carefully	3.58 ± 0.97
Identify tasks that should never be delegated	3.66 ± 0.96
Account impact of delegated tasks on the team	3.75 ± 1.05
Workload and staff schedule data before delegation	3.95 ± 0.86
Do not find delegation save time	3.59 ± 1.18
Think about tasks that should be delegated	3.74 ± 1.00
Delegate the work in a clear and concise manner	3.79 ± 0.99
Cannot delegate because staff lack experience	3.32 ± 1.10
When delegated, the head nurse loses control of the work	3.38 ± 1.21
Remarks staff, positive or negative, upon task completion	4.05 ± 0.73
Watch the time to end task	4.10 ± 0.97
Mean total delegation scale score (out of 150)	107.77 ± 13.27

*Note:* Detailed version of Table 2 is provided in the Appendix as supporting information (available here).

**Table 3:** Distribution of head nurses according to their degree of delegation as perceived by senior staff nurses (*N* = 112).

Degree of delegation	Frequency (*n*)	%
Never delegate	3	2.7
Sometimes delegate	67	59.8
Always delegate	42	37.5

**Table 4:** Senior staff nurses' perceived level of leadership styles of head nurses (*N* = 112).

Variables	Mean ± SD
Transformational leadership style	25.17 ± 7.31
I feel good when I am around my head nurse	1.97 ± 1.30
My head nurse provides me with new ways of looking at puzzling things	2.23 ± 1.10
My head nurse highlights the rewards available for what I accomplish	1.23 ± 1.38
I have full trust in my head nurse	1.98 ± 1.37
My head nurse gets me to rethink ideas that we had never questioned before	2.26 ± 1.12
My head nurse is satisfied when I meet agreed-upon standards	1.90 ± 1.28
I am proud to be associated with my head nurse	2.16 ± 1.15
My head nurse helps me to develop my selves	2.38 ± 1.05
As long as things are working, my head nurse does not try to change anything	2.05 ± 1.22
My head nurse expresses with a few simple words what we could and should do	2.20 ± 1.05
My head nurse informs me about their perspective on my performance	2.51 ± 1.03
My head nurse tells me the standards I need to know to carry out my work	2.29 ± 1.24
Transactional leadership style	12.97 ± 3.29
My head nurse provides appealing images about what we can do	2.25 ± 1.00
My head nurse gives personal attention to others who seem rejected	2.25 ± 1.16
My head nurse is content to let me continue working in the same way as always	2.16 ± 0.78
My head nurse helps me find meaning in my work	2.32 ± 1.01
My head nurse tells me what to do if I want to be rewarded for my work	1.61 ± 1.35
Whatever I want to do is okay with my head nurse	2.38 ± 0.84
Laissez-faire leadership style	5.66 ± 2.11
My head nurse enables me to think about old problems in new ways	2.23 ± 1.01
When I achieve my goals, my head nurse provides recognition or rewards	1.19 ± 1.31
No more is asked of me than what is essential by the head nurse	2.25 ± 0.89
Mean total leadership scale score (out of 110)	43.81 ± 10.90

*Note:* Detailed version of Table 4 is provided in the Appendix as supporting information (available here).

**Table 5:** Correlation between leadership styles and delegation practices of head nurses as perceived by senior staff nurses (*N* = 112).

Variables		1	2	3	4	5
1. Delegation	*r*	1				
	*p*					

2. Leadership style	*r*	0.290^∗∗^	1			
	*p*	0.002				

3. Transformational style	*r*	0.206^∗^		1		
	*p*	0.029				

4. Transactional style	*r*	0.321^∗∗^			1	
	*p*	0.001				

5. Laissez-faire style	*r*	0.279^∗∗^				1
	*p*	0.003				

^∗^ *p* < 0.05.

^∗∗^ *p* < 0.001.

**Table 6:** Multiple linear regression analysis predicting delegation practices from leadership styles of head nurses as perceived by senior staff nurses.

Dependent variable	Variables	*B*	SE	β	*t*	*p*	95% CI	
							Lower	Upper
Delegation practices	(Constant)	60.03	3.55		16.912	0.000	53.00	67.07
	Transformational leadership style	0.005	0.067	0.008	0.073	0.942	−0.129	0.138
	Transactional leadership style	0.153	0.073	0.238	2.109	0.037	0.009	0.29
	Laissez-faire leadership style	0.069	0.062	0.138	1.111	0.269	−0.054	0.19

Model summary	*F* (3108) = 4.75, *p*=0.004, *R*^2^ = 0.117							

*Note:* B = unstandardized coefficient; β = standardized coefficient; *p* < 0.05 indicates statistical significance; *R*^2^: adjusted *R*-squared value.

Abbreviation: SE = standard error.

## Data Availability

The data that support the findings of this study are available from the corresponding author upon reasonable request.

## Supporting Information

Appendix A. Detailed version of Table 2 is provided in the Appendix as supporting information.

Appendix B. Detailed version of Table 4 is provided in the Appendix as supporting information.
