# Supplementary material for: Leadership Styles and Delegation Practices of Head Nurses as Perceived by Senior Staff Nurses at a Hospital in Bangladesh
Source: J Nurs Manag. 2025 Sep 18;2025:5184762. doi: 10.1155/jonm/5184762 (PMC12463525; doi:10.1155/jonm/5184762)
Supplement: Supporting Information — Additional supporting information can be found online in the Supporting Information section. [file 5184762.f1.docx]

**Appendix A**

**Detailed version of Table 2 is provided in the Appendix as supplementary material.**

Table 2: Distribution of Head Nurses According to Their Agreement of Delegation as Perceived by Senior Staff Nurses (N=112)

| **Variables** | **Strongly Disagree** | | **Disagree** | | **Neutral** | | **Agree** | | **Strongly Agree** | | **Mean ± SD** |
| --- | --- | --- | --- | --- | --- | --- | --- | --- | --- | --- | --- |
|  | **f** | **(%)** | **f** | **(%)** | **f** | **(%)** | **f** | **(%)** | **f** | **(%)** |  |
| Delegate tasks to ease workload | 7 | 6.3 | 24 | 21.4 | 15 | 13.4 | 55 | 49.1 | 11 | 9.1 | 3.35 ± 1.11 |
| Show confidence in completing tasks | 2 | 1.8 | 17 | 15.2 | 25 | 22.3 | 55 | 49.1 | 13 | 11.6 | 3.54 ± 0.94 |
| Allow staff to undertake delegated work | 1 | 0.9 | 11 | 9.8 | 31 | 27.7 | 58 | 51.8 | 11 | 9.8 | 3.60 ± 0.83 |
| Know everyone’s strengths and weaknesses | 5 | 4.5 | 13 | 11.6 | 27 | 24.1 | 52 | 46.4 | 15 | 13.4 | 3.53 ± 1.01 |
| More precise than others | 10 | 8.9 | 14 | 12.5 | 14 | 12,5 | 54 | 48.2 | 20 | 17.9 | 3.54 ± 1.18 |
| No one can work as head nurse | 5 | 4,5 | 19 | 17.0 | 34 | 30.4 | 33 | 29.5 | 21 | 18.8 | 3.41 ± 1.11 |
| No one cares about work as head nurse | 3 | 2.7 | 18 | 16.1 | 33 | 29.5 | 39 | 34.8 | 19 | 17.0 | 3.47 ± 1.03 |
| Do not trust anyone about work secrets | 6 | 5.4 | 21 | 18.8 | 26 | 23.2 | 39 | 34.8 | 20 | 17.9 | 3.41 ±1,14 |
| Afraid if the head nurse delegates someone, he/she will hate the head nurse | 8 | 7.1 | 25 | 22.3 | 18 | 16.1 | 39 | 34.8 | 22 | 19.6 | 3.38 ±1.23 |
| Delegation opportunity to develop individuals | 4 | 3.6 | 9 | 8.0 | 14 | 12.5 | 64 | 57.1 | 21 | 18.8 | 3.79 ± 0.96 |
| Provide training to inexperience staff | 8 | 7.1 | 14 | 12.5 | 17 | 15.2 | 46 | 41.1 | 27 | 24.1 | 3.63 ± 1.18 |
| Supervise every detail of staff work | 4 | 3.6 | 9 | 8.0 | 14 | 12.5 | 64 | 57.1 | 21 | 18.8 | 3.79 ± 0.96 |
| Tell staff their authority when delegating the task | 6 | 5.4 | 18 | 16.1 | 14 | 12.5 | 51 | 45.5 | 23 | 20.5 | 3.60 ± 1.14 |
| Can not take a vacation, work will collapse | 9 | 8.0 | 35 | 31.3 | 18 | 16.1 | 38 | 33.9 | 12 | 10.7 | 3.08 ± 1.18 |
| Tolerate mistakes | 9 | 8.0 | 17 | 15.2 | 24 | 21.4 | 53 | 47.3 | 9 | 8.0 | 3.32 ± 1.08 |
| Balance the workload of staff | 4 | 3.6 | 16 | 14.3 | 20 | 17.9 | 58 | 51.8 | 14 | 12.5 | 3.55 ± 1.00 |
| Watch the work of staff and correct mistakes | 7 | 6.3 | 13 | 11.6 | 12 | 10.7 | 59 | 52.7 | 21 | 18.8 | 3.66 ± 1.10 |
| Find myself compelled to re-work | 6 | 5.4 | 21 | 18.8 | 20 | 17.9 | 51 | 45.5 | 14 | 12.5 | 3.41 ± 1.09 |
| Delegate all jobs, review the results | 3 | 2.7 | 7 | 6.3 | 23 | 20.5 | 54 | 48.2 | 25 | 22.3 | 3.81 ±0.94 |
| Staff lack commitment, if delegated, work will not be implemented carefully | 3 | 2.7 | 16 | 14.3 | 20 | 17.9 | 59 | 52.7 | 14 | 12.5 | 3.58 ± 0.97 |
| Identify tasks that should never be delegated | 3 | 2.7 | 15 | 13.4 | 14 | 12.5 | 65 | 58.4 | 15 | 13.4 | 3.66 ± 0.96 |
| Account impact of delegated tasks on the team | 6 | 5.4 | 11 | 9.8 | 10 | 8.9 | 62 | 55.4 | 23 | 20.5 | 3.75 ± 1.05 |
| Workload & staff schedule data before delegation | 1 | 0.9 | 7 | 6.3 | 18 | 16.1 | 57 | 50.9 | 29 | 25.9 | 3.95 ± 0.86 |
| Do not find delegation save time | 4 | 3.6 | 19 | 17.0 | 29 | 25.9 | 27 | 24.1 | 33 | 29.5 | 3.59 ± 1.18 |
| Think about tasks that should be delegated | 1 | 0.9 | 16 | 14.3 | 20 | 17.9 | 49 | 43.8 | 26 | 23.2 | 3.74 ± 1.00 |
| Delegate the work in a clear & concise manner | 5 | 4.5 | 6 | 5.4 | 21 | 18.8 | 55 | 49.1 | 25 | 22.3 | 3.79 ± 0.99 |
| Can not delegate because staff lack experience | 4 | 3.6 | 30 | 26.8 | 18 | 16.1 | 46 | 41.1 | 14 | 12.5 | 3.32 ± 1.10 |
| When delegated, the head nurse loses control of the work | 6 | 5.4 | 28 | 25.0 | 18 | 16.1 | 37 | 33.0 | 23 | 20.5 | 3.38 ± 1.21 |
| Remarks staff, positive or negative, upon task completion | 2 | 1.8 | 5 | 4.5 | 8 | 8.0 | 70 | 62.5 | 27 | 24.1 | 4.05 ± 0.73 |
| Watch the time to end task | 5 | 4.5 | 5 | 4.5 | 3 | 2.7 | 60 | 53.6 | 39 | 34.8 | 4.10 ± 0.97 |

**Appendix B**

**Detailed version of Table 4 is provided in the Appendix as supplementary material.**

Table 4: Distribution of Senior Staff Nurses’ Perception of Leadership Styles of Head Nurses (N=112)

| **Variables** | **Not at all** | | **Once in a while** | | **Sometimes** | | **Fairly often** | | **Frequently, if not always** | | **Mean ±SD** |
| --- | --- | --- | --- | --- | --- | --- | --- | --- | --- | --- | --- |
|  | **f** | **(%)** | **f** | **(%)** | **f** | **(%)** | **f** | **(%)** | **f** | **(%)** |  |
| **Transformational Leadership Style** | | | | | | | | | | | |
| I feel good when I am around my head nurse. | 23 | 20.54 | 12 | 10.71 | 37 | 33.04 | 25 | 22.32 | 15 | 13.39 | 1.97 ± 1.30 |
| My head nurse provides me with new ways of looking at puzzling things. | 8 | 7.14 | 20 | 17.86 | 36 | 32.14 | 34 | 30.36 | 14 | 12.50 | 2.23 ± 1.10 |
| My head nurse highlights the rewards available for what I accomplish. | 53 | 47.32 | 14 | 12.50 | 20 | 17.86 | 16 | 14.29 | 9 | 8.04 | 1.23 ± 1.38 |
| I have full trust in my head nurse. | 23 | 20.54 | 18 | 16.07 | 28 | 25.00 | 24 | 21.43 | 19 | 16.96 | 1.98 ± 1.37 |
| My head nurse gets me to rethink ideas that we had never questioned before. | 11 | 9.82 | 11 | 9.82 | 44 | 39.29 | 30 | 26.79 | 16 | 14.29 | 2.26 ± 1.12 |
| My head nurse is satisfied when I meet agreed-upon standards. | 24 | 21.43 | 15 | 13.39 | 31 | 27.68 | 32 | 28.57 | 10 | 8.93 | 1.90 ± 1.28 |
| I am proud to be associated with my head nurse. | 12 | 10.71 | 16 | 14.29 | 40 | 35.71 | 30 | 26.79 | 14 | 12.50 | 2.16 ± 1.15 |
| My head nurse helps me to develop my selves. | 6 | 5.36 | 15 | 13.39 | 36 | 32.14 | 40 | 35.71 | 15 | 13.39 | 2.38 ± 1.05 |
| As long as things are working, my head nurse does not try to change anything. | 15 | 13.39 | 23 | 20.54 | 28 | 25.00 | 33 | 29.46 | 13 | 11.61 | 2.05 ± 1.22 |
| My head nurse expresses with a few simple words what we could and should do. | 4 | 3.57 | 27 | 24.11 | 38 | 33.93 | 29 | 25.89 | 14 | 12.50 | 2.20 ± 1.05 |
| My head nurse informs me about their perspective on my performance. | 4 | 3.57 | 13 | 11.61 | 37 | 33.04 | 38 | 33.93 | 20 | 17.86 | 2.51 ± 1.03 |
| My head nurse tells me the standards I need to know to carry out my work. | 11 | 9.82 | 21 | 18.75 | 26 | 23.21 | 33 | 29.46 | 21 | 18.75 | 2.29 ± 1.24 |
| **Transactional Leadership Style** | | | | | | | | | | | |
| My head nurse provides appealing images about what we can do. | 5 | 4.46 | 20 | 17.86 | 39 | 34.82 | 38 | 33.93 | 10 | 8.93 | 2.25 ± 1.00 |
| My head nurse gives personal attention to others who seem rejected. | 13 | 11.61 | 12 | 10.71 | 35 | 31.25 | 38 | 33.93 | 14 | 12.50 | 2.25 ± 1.16 |
| My head nurse is content to let me continue working in the same way as always. | 5 | 4.46 | 7 | 6.25 | 70 | 62.50 | 25 | 22.32 | 5 | 4.46 | 2.16 ± 0.78 |
| My head nurse helps me in finding meaning in my work. | 6 | 5.36 | 16 | 14.29 | 37 | 33.04 | 42 | 37.50 | 11 | 9.82 | 2.32 ± 1.01 |
| My head nurse tells me what to do if I want to be rewarded for my work. | 34 | 30.36 | 19 | 16.96 | 28 | 25.00 | 19 | 16.96 | 12 | 10.71 | 1.61 ± 1.35 |
| Whatever I want to do is okay with my head nurse. | 2 | 1.79 | 9 | 8.04 | 56 | 50.00 | 34 | 30.36 | 11 | 9.82 | 2.38 ± 0.84 |
| **Laissez-fair Leadership Style** | | | | | | | | | | | |
| My head nurse enables me to think about old problems in new ways. | 8 | 7.14 | 12 | 10.71 | 49 | 43.75 | 32 | 28.57 | 11 | 9.82 | 2.23 ± 1.01 |
| When I achieve my goals, my head nurse provides recognition or rewards. | 53 | 47.32 | 12 | 10.71 | 28 | 25.00 | 11 | 9.82 | 8 | 7.14 | 1.19 ± 1.31 |
| No more is asked to me than what is absolutely essential by head nurse. | 6 | 5.36 | 7 | 6.25 | 61 | 54.46 | 29 | 25.89 | 9 | 8.04 | 2.25 ± 0.89 |
